# Proteomics analysis of aqueous and vitreous humor in uveitis: a systematic literature review

**DOI:** 10.1186/s12014-025-09564-2

**Published:** 2025-12-16

**Authors:** Susanne Reeg, Oliver Niels Klefter, Yousif Subhi, Henrik Vorum, Bent Honoré, Lasse Jørgensen Cehofski

**Affiliations:** 1 Department of Ophthalmology, Odense University Hospital, Odense, Denmark; 2 Department of Ophthalmology, Rigshospitalet, Glostrup, Danmark; 3 Department of Clinical Medicine, University of Copenhagen, Copenhagen, Denmark; 4 Department of Clinical Research, University of Southern Denmark, Odense, Denmark; 5 Department of Ophthalmology, Aalborg University Hospital, Aalborg, Denmark; 6 Department of Clinical Medicine, Aalborg University, Aalborg, Denmark; 7 Department of Biomedicine, Aarhus University, Aarhus, Denmark

**Keywords:** Uveitis, Mass spectrometry, Proteomics, Aqueous humor, Vitreous, Ocular inflammation, Intraocular

## Abstract

**Purpose:**

Uveitis is an inflammatory ocular disease with diverse etiologies and pathogeneses. It potentially leads to significant visual impairment and socioeconomic burden. Proteomic analysis can provide insights into protein-driven mechanisms that may improve diagnosis, monitor disease progression, and identify therapeutic targets. Here, we summarize the proteomic results from studies investigating the aqueous and vitreous humor in eyes with uveitis versus non-inflammatory controls.

**Methods:**

A comprehensive search of 15 databases was conducted on January 26, 2024. Studies were included if they performed proteomic analyses using mass spectrometry on aqueous or vitreous humor from uveitis patients. The selection, data extraction, and risk of bias assessment were performed independently by multiple reviewers, with a third reviewer consulted in case of disagreement. Six studies met the eligibility criteria, comprising 176 eyes of uveitis patients and 105 control eyes.

**Results:**

Two proteins, complement C1q subcomponent subunit B and C1q subcomponent subunit C, were consistently upregulated in five studies, underscoring the role of complement activation in uveitis pathogenesis. Three additional proteins − alpha-2-HS-glycoprotein, apolipoprotein A-I, and alpha-1-antichymotrypsin − were upregulated in four studies, highlighting the significance of inflammatory modulation. Ceruloplasmin, an acute-phase reactant, was upregulated in four studies. Gelsolin kininogen-1, and alpha-1-antitrypsin were upregulated in three studies, indicating a pro-inflammatory shift towards increased vascular permeability and recruitment of inflammatory cells.

**Conclusion:**

The identified proteome changes highlight central biological processes in uveitis, notably complement activation, acute-phase response, pro-inflammatory shift, and increased vascular permeability. The identified proteins can potentially support future diagnostic and therapeutic advances in uveitis.

**Supplementary Information:**

The online version contains supplementary material available at 10.1186/s12014-025-09564-2.

## Introduction

Uveitis is an inflammatory ocular disease with highly heterogeneous etiologies and pathogenic mechanisms [1]. It is potentially sight-threatening and includes diverse conditions of inflammation of the uvea. The retina, optic disc, vitreous humor (VH), and other surrounding tissues can also be involved [2]. The associated visual impairment causes a significant burden, reducing patients’ overall quality of life and adversely impairing their socioeconomic conditions [3]. The high prevalence of uveitis in working-age populations leads to high expenses related to healthcare costs and workforce absence [4]. Furthermore, uveitis may be the first sign of various severe systemic conditions [5].

The incidence and prevalence of uveitis depend on factors, such as geographical location, age, gender, social habits, etiology, and genetics [5]. The estimated annual incidence of uveitis is 50.45 per 100,000 with a prevalence ranging from 9 to 730 cases per 100,000 [6]. Uveitis accounts for 10–20% of blindness in the United States and Europe and possibly up to 25% in other parts of the world. Likewise, the anatomical distribution and infectious versus non-infectious etiologies vary between populations [4, 5, 7–26].

The comprehensive analysis of proteins in ocular fluids offers the possibility to elucidate variances in pathophysiological pathways of distinct uveitis subtypes. Significantly, it may detect proteins that could be used to enhance diagnostic accuracy, monitor disease progression, or discover prospective therapeutic targets [27].

"Proteome" describes the complete array of proteins within a specific tissue. As a concept, proteomics aims to systematically identify and quantify the entirety of proteins present in a specific tissue sample or bodily fluid.

The field of proteomics is constantly evolving. New proteomic profiling methods are being developed, providing increasing depth in protein coverage, thereby allowing a deeper understanding of disease mechanisms. Mass spectrometry-based and affinity-based approaches are the two major techniques for proteomic profiling. This review focuses on mass spectrometry-based techniques as they allow for unbiased large-scale protein analysis that are not limited to a specific set of proteins that are defined beforehand. The proteomic analysis is based on a multi-step workflow where key proteins identified by mass spectrometry are selected for further validation by other quantitative techniques such as ELISA, Western blotting, or targeted mass spectrometry, namely either multiple reaction monitoring (MRM) or selective reaction monitoring. The reader is directed to previous publications for in-depth reviews of ocular proteomics [28].

Proteome studies of uveitis often operate with small sample sizes. Therefore, a systematic analysis and synthesis of the evidence will be of significant relevance. In this study, we reviewed the literature systematically, to give a concise summary of the most consistent protein alterations in uveitis and their biological implications.

## Methods

This study was a systematic review. Our study protocol was registered in the PROSPERO database (CRD42024516805). Dissemination of this study followed the principles of the PRISMA statement [29].

### Eligibility criteria

To be included in this review, studies had to meet the following eligibility criteria:

### Study population

The study group had to include human eyes diagnosed with uveitis, with no restrictions on age or the specific type and severity of uveitis.

### Control group

Control groups must exclude patients with diabetic retinopathy, maculopathy, or retinal vascular occlusions, as these conditions are known to influence intraocular levels of pro- or anti-inflammatory proteins [30–33]. The control group should consist of human eyes that were either healthy or have non-inflammatory conditions. Acceptable control conditions included eyes undergoing pars plana vitrectomies for issues such as epiretinal membrane, macular hole, surgery for cataract or glaucoma.

### Proteomic analysis

Eligible studies needed to report results from proteomic analyses of the aqueous (AH) and/or VH. Only studies employing mass spectrometry for proteomic analysis and presenting original data were included.

### Outcomes

There were no restrictions on the outcomes reported by the individual studies.

### Language and publication

Only studies published in English were considered. There were no geographical or journal-specific restrictions.

### Information sources and search strategy

On 26 January 2024, a comprehensive literature search was conducted across multiple databases. The databases searched included PubMed, Cochrane, Embase, Web of Science Core Collection, BIOSIS Previews, Current Contents Connect, Data Citation Index, Derwent Innovations Index, Grants Index, KCI-Korean Journal Database, MEDLINE, Preprint Citation Index, ProQuest Dissertations & Theses Citation Index, SciELO Citation Index, and Zoological Record. The search terms were developed by trained individuals (O.K. and Y.S.) and were customized for each database. Detailed search phrases and the results from each database are provided in Appendix S1.

### Study selection, data collection, and risk of bias assessment

One author (SR) screened the titles and abstracts from the search results, removing duplicates and clearly irrelevant studies. Subsequently, two authors (S.R. and L.J.C.) independently reviewed the full texts of the remaining studies to determine eligibility, and they also examined the references of these studies for any additional relevant studies. Data extraction from eligible studies, focusing on study design, characteristics, methods, and results, was performed independently by authors S.R. and L.J.C. using a standardized extraction form. In cases of disagreement during study selection and data extraction, a third author (O.K.) was consulted to achieve consensus.

Given that all included studies were cross-sectional comparisons of two or more groups, the risk of bias was assessed using selected items from the Agency for Healthcare Research and Quality Checklist (Q1-Q4, Q6, Q7), as recommended for such studies [34]. This assessment was conducted independently by two authors (S.R. and Y.S.). Their results were compared, and any discrepancies were resolved through discussion. If consensus could not be reached, a third author (O.K.) was involved to finalize the decision.

### Outcome measures, data analysis, and synthesis

The primary outcome of interest was the differences in protein levels in the AH or VH between eyes with uveitis versus eyes without any history of ocular inflammation. The outcomes were not reported in a manner conducive to quantitative analysis, and meta-analysis was not feasible due to the heterogeneity of the included studies. Therefore, the studies were reviewed qualitatively and summarized in tables. Proteins identified in more than one study were listed in a separate table to show consistent findings, potentially indicating general proteomic differences in the AH or VH between patients with uveitis and controls.

## Results

### Study selection

**Fig. 1:**
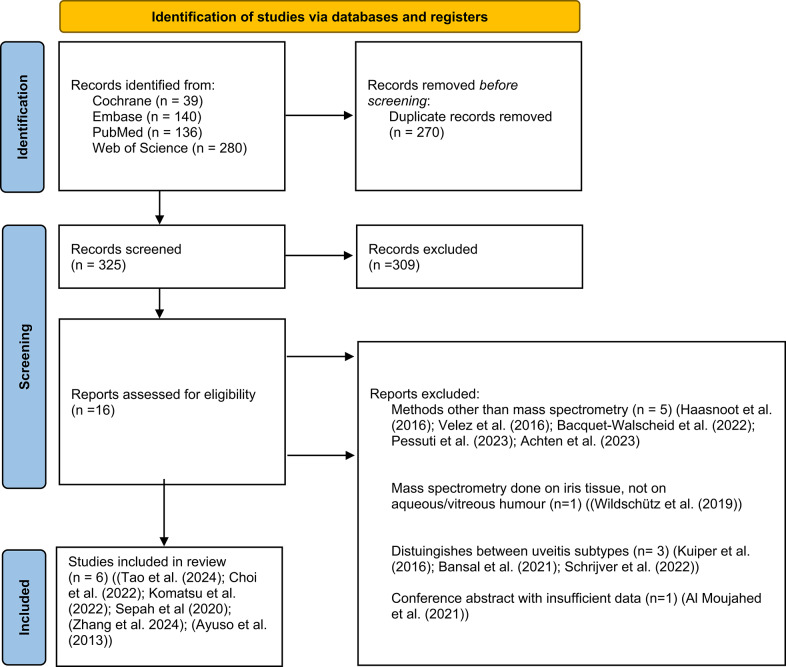
Flow diagram of study selection following the PRISMA 2020 guidelines. Adapted from: Page MJ, McKenzie JE, Bossuyt PM, Boutron I, Hoffmann TC, Mulrow CD, et al. The PRISMA 2020 statement: an updated guideline for reporting systematic reviews. BMJ. 2021;372:n71

The literature search yielded 595 records, with 270 identified as duplicates. Of the remaining 325 records, 16 passed the abstract screening and were reviewed in full text. Six records met the eligibility criteria and were included in this review (Fig. 1). Details on the literature search can be found [see Supplement 1].

### Study design characteristics of eligible studies

All eligible studies had a cross-sectional design. They compared a study group to a control group, either in the main analysis or in a subanalysis. The characteristics of the reviewed studies are summarized in Table 1. One of the studies [41] included two study groups, each compared separately to controls, while another study [35] had three study groups, also compared separately to controls. The other four articles had one study group each, which was compared to controls [36–39]. All studies taken together, the study group consisted of 176 eyes of 176 patients and the control group consisted of 105 eyes of 105 patients. Overall, the individual study groups were small, ranging from 4 to 39 eyes. There was a broad range in mean ages across the studies, both within the study groups and control groups. In five studies, the mean age range for study groups spanned from 26.5 to 69.5 years, and for control groups from 61.3 to 65.8 years [36–39, 41]. A study including children [35] had a mean age of 11.1 to 13.8 years in its study subgroups and a mean age of 4.2 years in the control group.

**Table 1:** Characteristics of studies included in the systematic review

**Reference**	**Study group**		**Control group**		**Sampling**	**Proteomic analysis**	**Found differentially expressed proteins**
	**Clinical definition**	**Demographics**	**Clinical definition**	**Demographics**			
Ayuso et al. [35]	JIA uveitis (n = 14) Silent chronic AU (n = 8) Other uveitis (n = 31)	JIA uveitis: age 11.5 (4.9–23.9); 4:10 (Male: Female) Silent chronic AU: age 11.1 (8.1–16.6); 2:6 (Male: Female) Other uveitis: age 13.8 (0.1–17.8); 15:16 (Male: Female)	congenital cataract (n = 11), traumatic cataract (n = 3), congenital glaucoma (n = 6)	Age 4.2 (0.4–15.9) 12:8 (Male: Female)	Study group: AH collected during therapeutically necessary intraocular surgery or for diagnostic purposes Controls: AH collected during elective intraocular surgery	SELDI-ToF MS and LC–MS/MS	JIA vs controls (SELDI-ToF MS) 6 protein peaks were significantly differentially expressed JIA and silent chronic AU vs. other uveitis entities and controls (LC–MS/MS) The protein at m/z 13,762 was identified as TTR
Sepah et al. [36]	IU (n = 4)	Age 26.0 ± 9.2 years 1:3 (Male: Female)	Idiopathic macular hole (n = 4)	Age 62 ± 5.0 years 2:2 (Male: Female)	Liquid vitreous collected under pars plana vitrectomy	LC–MS/MS	IU vs controls 233 proteins DEPs 103 upregulated, 130 downregulated
Komatsu et al. [37]	Ocular sarcoidosis (n = 10)	Age 69.5 ± 10.1 years 2:8 (Male: Female)	epiretinal membrane or macular hole (n = 10)	Age 62.1 ± 13.3 years 5:5 (Male: Female)	Liquid vitreous collected under pars plana vitrectomy	LC–MS/MS	Ocular sarcoidosis vs controls 290 proteins were DEPs 263 upregulated, 27 downregulated
Choi et al. [38]	CMV-HAU (n = 10)	Age 53.1 ± 15.3 years 8:2 (Male: Female)	Cataract (n = 10)	Age 65.8 ± 6.9 years 4:6 (Male: Female)	Study group: AH obtained by anterior chamber tab Controls: AH obtained before surgical procedure for cataract	LC–MS/MS	CMV-HAU vs controls 65 proteins were DEPs 50 upregulated, 15 downregulated
Tao et al. [39]	FUS (n = 15)	Age 37.3 ± 13.93 years 9/6 (Male: Female)	Age-related cataract (n = 16)	Age 61.3 ± 5.8 years 7/9 (Male: Female)	AH obtained before surgical procedure for cataract	LC–MS/MS a triple time-of-flight (TOF) 6600 mass spectrometer	FUS vs controls 174 proteins were DEPs 78 upregulated, 96 downregulated
Zhang et al. [40, 41]	VKH Vogt-Koyanagi-Harada uveitis (n = 45)	Age 49.56 ± 10.67 years 30/15 (Male: Female)	senile cataracts (n = 45)	Age 64.78 ± 9.72 years 30/15 (Male: Female)	AH obtained by anterior chamber paracentesis, at the beginning of the cataract surgery	LC–MS/MS AH exosomes (pooled samples by mixing six AH samples from six participants matched by age and gender in each group	VKH group vs control 65 proteins were DEPs 40 up-regulated, 25 down-regulated
	BU (n = 39)	Age 40.00 ± 8.47 years 30/9 (Male: Female)					BU vs control 40 proteins were DEPs 38 up-regulated, 2down-regulated proteins

Age reported in mean ± standard deviation; AH = aqueous humour; BU = Behcet’s uveitis; CMV-HAU = CMV hypertensive anterior uveitis; DEPs = differentially expressed proteins; FUS = Fuchs uveitis syndrome; HR-MRM = high resolution multiple reaction monitoring; IU = intermediate uveitis; JIA uveitis = juvenile idiopathic arthritis uveitis; LC–MS/MS = liquid chromatography-tandem mass spectrometry; silent chronic AU = silent chronic anterior uveitis; transthyretin = TTR; VKH uveitis = Vogt-Koyanagi-Harada uveitis;

Concerning gender distribution, three of the six studies had more female subjects than males in the study groups [35–37]. The remaining studies had more male subjects in the study groups [38, 39, 41]. In the control groups, two studies reported a balanced gender distribution [36, 37], two had more females [38, 39], and two had more males [35, 41].

Five studies were published between 2020 and 2024 [36–39, 41]. One study was published in 2013 [35].

### Characteristics of the study groups and the control groups

The studies included in our systematic review had diverse cohorts of uveitis-afflicted eyes as study groups. Ayuso et al. [35] divided their study population into four groups: (1) eyes with juvenile idiopathic arthritis-associated uveitis, (2) silent chronic anterior uveitis, (3) a group consisting of various other forms of uveitis, including symptomatic anterior, intermediate, posterior, and panuveitis with diverse etiologies and (4) a control group. A comprehensive comparison of the proteome of the AH of all four groups was made. Additionally, the AH proteome of eyes of juvenile idiopathic arthritis-associated uveitis was compared separately to the proteome of the AH of each of the other groups [35]. Zhang et al. had two separate study groups: eyes affected by Vogt-Koyanagi-Harada (VKH) uveitis and Behcet’s uveitis. They compared the proteome of AH-derived exosomes from these groups with those of a control group [41]. Choi et al. compared the proteome of AH from eyes with cytomegalovirus-associated hypertensive anterior uveitis (CMV-HAU) to that of controls [38]. Sepah et al. had eyes with idiopathic intermediate uveitis as study group and compared the proteome of their liquid VH to that of controls [36]. Tao et al. examined the proteome of AH from eyes with Fuchs’ uveitis syndrome in comparison to controls [39]. Komatsu et al. had eyes with ocular sarcoidosis as study group and compared the proteome of their liquid VH to controls [37].

The included studies’ control groups all consisted of eyes diagnosed with non-inflammatory conditions. In Komatsu et al., the control group consisted of eyes diagnosed with epiretinal membrane or macular holes [37]. Sepah et al., only had eyes with idiopathic macular holes in their control group [36]. Additionally, three studies included eyes with cataracts as control groups, while Ayuso et al. [35] included a control group consisting of eyes diagnosed with either pediatric cataracts or glaucoma.

### Methods employed for sampling and analysis

Liquid VH samples were collected during pars plana vitrectomy, as performed in two studies [36, 37]. Another study collected AH samples during therapeutically necessary intraocular surgeries or for diagnostic purposes, while control samples were collected during elective intraocular surgeries [35]. AH samples were collected by anterior chamber tap or paracentesis at the beginning of cataract surgery in some studies [38, 39, 41]. For proteomic analysis, all studies employed liquid chromatography coupled with tandem mass spectrometry (LC–MS/MS). Specifically, one study used Surface-Enhanced Laser Desorption/Ionization Time of Flight Mass Spectrometry (SELDI-ToF MS) to find protein peaks, measured in mass–charge ratio and only identified the protein of one of those peaks with the help of LC–MS/MS [35] while another focused on analyzing AH exosomes using LC–MS/MS [41].

Four of six studies included in this review used Enzyme-Linked Immunosorbent Assay (ELISA) or High-Resolution Multiple Reaction Monitoring (HR-MRM) to validate their most important proteomic findings. Two of those four used ELISA to confirm the presence of significant proteins identified through mass spectrometry [35, 37]. The third study used HR-MRM and Western blotting for validation [39]. The fourth study used Western blotting only to confirm their findings [41]. An overview of the validated proteins is shown in Table 2.

**Table 2:** Proteins discovered with mass spectrometry and validated with ELISA, HR-MRM or Western blotting

Protein ID	Protein name	Functions	Level in uveitis relative to controls	Method	Study
P80188	Neutrophil gelatinase-associated lipocalin (NGAL)	Apoptotic process defence response to bacterium innate immune response positive regulation of cold-induced thermogenesis siderophore transport	In sarcoidosis increased compared to VRL, controls, and BU	ELISA	Komatsu et al. [37]
P57087	Junctional adhesion molecule B (JAM-B)	Involved in various cell adhesion processes, migration of cells (such as leukocytes and hematopoietic stem cells), maintaining the blood–brain barrier, myelination regulation, and development of sperm cells	In sarcoidosis increased significantly compared to VRL and controls, but no significant difference compared to BU	ELISA	Komatsu et al. [37]
P05060	Secretogranin-1 (CHGB)	Regulation of Hormone and Neurotransmitter Packaging Facilitation Secretion Process Maintenance of Cellular Homeostasis	↓	HR-MRM	Tao et al. [39]
P02746	C1QB	Complement activation, specifically classical pathway Innate immune response Synapse pruning	↑	HR-MRM; Western blotting	Tao et al. [39], Zhang et al. [40, 41]
P00450	Ceruloplasmin (CP)	Transport of copper and iron ions and maintenance of iron homeostasis within cells	↑	Western blotting	Zhang et al. [40, 41]
P02766	Transthyretin (TTR)	Transport of thyroid hormones and retinol-binding protein	↑	ELISA	Ayuso et al. [35]

Behçet uveitis = BU; ELISA = Enzyme-Linked Immunosorbent Assay; HR-MRM = high resolution multiple reaction monitoring; Vitreoretinal lymphoma = VRL

### Global intraocular proteome changes associated with uveitis

In all studies, p-values less than 0.05 were considered statistically significant. The threshold for fold change differed across the studies. False discovery rates also had varying thresholds. Significant proteomic changes in uveitis were observed in all studies when compared to controls. There was an overlap in the protein changes identified across different studies: 112 proteins were significantly up- or downregulated in two or more studies, as listed in Table 3. More data and filters are available [see Supplement 2]. Among these, 70 proteins were consistently upregulated in two or more studies, 31 proteins showed inconsistent regulation (either up- or downregulated), and 11 proteins were downregulated in two studies. Notably, no proteins were downregulated in three or more studies.

**Table 3:** Overview of proteins identified in eyes with uveitis in two or more independent studies

**Protein**	Choi et al. [38]	Zhang et al. [40, 41]**—BU **	Zhang et al. [40, 41]**—VKH**	Komatsu et al. [37]	Tao et al. [39]	Ayuso et al. [35]	**Total number of studies reporting differential expression**	**↑ Upregulated** **↓ Downregulated** **↑↓ Conflicting studies**
A1BG			1	1			2	**↑**
ABI3BP				−1	−1		2	**↓**
ACTB				1	1		2	**↑**
ADA2				1	1		2	**↑**
AFM	1			1			2	**↑**
AGT	1			1			2	**↑**
AHSG		1	1	1	1		4	**↑**
ALB	1	1	1				3	**↑**
ALDOA				1	1		2	**↑**
ALDOC				1	1		2	**↑**
ANG				1	−1		2	**↑↓**
APOA1	1	1	1	1			4	**↑**
APOA2		1	1		1		3	**↑**
APOB				1	1		2	**↑**
APOC1		1	1				2	**↑**
APOE	1	1					2	**↑**
AZGP1				1	1		2	**↑**
B2M				1	1		2	**↑**
BLMH		1	1				2	**↑**
C1QA			1	1	1		3	**↑**
C1QB	1	1	1	1	1		5	**↑**
C1QC	1	1	1	1	1		5	**↑**
C1R				1	1		2	**↑**
C1S			−1	1	1		3	**↑↓**
C3		1			−1		2	**↑↓**
C4B	1			1	1		3	**↑**
C5	1				−1		2	**↑↓**
C7	1			1			2	**↑**
CD14	1			1	1		3	**↑**
CD163				1	1		2	**↑**
CD44				1	−1		2	**↑↓**
CD59			−1		−1		2	**↓**
CFB	1			1	1		3	**↑**
CFD	1			1			2	**↑**
CFI	1			1	−1		3	**↑↓**
CHI3L1				1	1		2	**↑**
CLU	1	1			1		3	**↑**
CP	1	1	1	1	−1		5	**↑↓**
CSF1R				1	1		2	**↑**
ECM1	−1			1			2	**↑↓**
EFEMP1		1			−1		2	**↑↓**
ENO1				1	1		2	**↑**
F13B				1	1		2	**↑**
F5	1				−1		2	**↑↓**
FCGR3A				1	1		2	**↑**
FGA	1			1			2	**↑**
FGB		1	1				2	**↑**
FGG		1	1				2	**↑**
FLNA			−1	1			2	**↑↓**
FN1				1	1		2	**↑**
FSTL1	−1				−1		2	**↓**
GAPDH	1			1			2	**↑**
GC				1	−1		2	**↑↓**
GNS				−1	−1		2	**↓**
GSN	1	1		1	−1		4	**↑↓**
HPX		1	1				2	**↑**
HRG	1	1			1		3	**↑**
HSPG2	1				−1		2	**↑↓**
ICOSLG				1	−1		2	**↑↓**
IFI30				1	1		2	**↑**
IGA2		1	1				2	**↑**
IGFBP6	−1			1			2	**↑↓**
IGG1		1	1				2	**↑**
IGHA1		1	1				2	**↑**
IGHG3		1	1		−1		3	**↑↓**
IGHM			1		1		2	**↑**
IGHV3-15	1				−1		2	**↑↓**
IGKC		1	1				2	**↑**
IGKV3-20			1		1		2	**↑**
IGLC2		1	1				2	**↑**
ITIH1	1		−1				2	**↑↓**
JCHAIN		1	1		1		3	**↑**
KNG1	1		1	1	−1		4	**↑↓**
KRT16		−1	−1				2	**↓**
LAP3				1	1		2	**↑**
LCP1				1	1		2	**↑**
LUM	−1				−1		2	**↓**
MAN1A1				1	−1		2	**↑↓**
MFAP2		1	1				2	**↑**
MMP9				1	1		2	**↑**
OPTC			−1		1		2	**↑↓**
PFN1				1	1		2	**↑**
PGK1				1	1		2	**↑**
PHB2		1	1				2	**↑**
PROC				1	−1		2	**↑↓**
PTGDS		1	1		−1		3	**↑↓**
RBP4	1			1			2	**↑**
RELN	−1				−1		2	**↓**
RPL19		1	1				2	**↑**
S100A9				1	1		2	**↑**
SECTM1				1	1		2	**↑**
SERPINA1	1	1	1		−1		4	**↑↓**
SERPINA3	1	1	1	1			4	**↑**
SERPINA4				1	−1		2	**↑↓**
SERPINA5				1	−1		2	**↑↓**
SERPINA6				1	−1		2	**↑↓**
SERPINA7	1			1			2	**↑**
SERPINB1				1	1		2	**↑**
SERPINC1	1				−1		2	**↑↓**
SERPIND1	1				−1		2	**↑↓**
SERPINF1		1	1				2	**↑**
SERPINI1	1			−1	−1		3	**↑↓**
SPARCL1	−1				−1		2	**↓**
SPOCK1				−1	−1		2	**↓**
TF		1	1				2	**↑**
TPI1	1	1					2	**↑**
TPP1				−1	−1		2	**↓**
TTR				1		1	2	**↑**
VIM				1	1		2	**↑**
VSIG4				1	1		2	**↑**
WIF1				−1	−1		2	**↓**
YWHAE				1	1		2	**↑**

1 = up-regulated

−1 = down-regulated

Two proteins − complement C1q subcomponent subunit B and complement C1q subcomponent subunit C − were consistently upregulated in five studies. Three proteins—alpha-2-hs-glycoprotein, apolipoprotein a-i, and alpha-1-antichymotrypsin (ACT) − were upregulated in four studies. Ceruloplasmin was upregulated in four studies but downregulated in the study by Tao et al. [39]. Similarly, three proteins— Gelsolin (AGEL), Kininogen-1 (Alpha-2-thiol proteinase inhibitor), and Alpha-1-antitrypsin − were upregulated in three studies but downregulated in the study by Tao et al. [39]. Nine proteins—albumin, apolipoprotein a-II, complement c1q subcomponent subunit a, complement c4-b, monocyte differentiation antigen CD12, complement factor b, clusterin, histidine-rich glycoprotein and immunoglobulin j chain—were upregulated in three studies without any instances of downregulation, while five proteins were either up- or downregulated in three studies.

Six proteins were validated using Western blotting, HR-MRM, or ELISA techniques: transthyretin (TTR), neutrophil gelatinase-associated lipocalin (NGAL), junctional adhesion molecule b (JAM-B), secretogranin-1 (CHGB), c1qb, and ceruloplasmin.

For all other proteins that were significantly up- or downregulated in only a limited number of studies, the supplementary material provides a description of their functions in the context of uveitis and other ophthalmologic conditions [see Supplement 3].

### Ocular sarcoidosis

Komatsu et al. [37]. performed a proteome analysis on the VH humor of eyes affected by ocular sarcoidosis and control samples consisting of VH samples from eyes with epiretinal membrane or macular hole.

The study identified 263 upregulated proteins and 27 downregulated proteins. Neutrophil granulation, associated with the innate immune system, was the most significantly upregulated pathway in ocular sarcoidosis. Notably, eight out of the top ten pathways were part of the broader immune system pathway. Among downregulated pathways, the most pronounced downregulation was observed in collagen chain trimerization.

### Fuchs uveitis syndrome

Tao et al. [39]. compared the proteome of the AH from eyes with Fuchs uveitis syndrome to the proteomes of age-related cataract and Posner-Schlossman syndrome.

Regulated pathways in Fuchs uveitis vs. controls included hypoxia-inducible factor-1 (HIF-1) signaling, acute inflammatory response, humoral immune response, response to bacterium, viral infection, FCyR-mediated phagocytosis, and complement and coagulation cascades.

In the comparative analysis of the differential proteomic profiles between Fuchs uveitis syndrome and Posner-Schlossman syndrome, 50 upregulated and 21 downregulated differentially expressed proteins were identified. In Fuchs uveitis compared to Posner-Schlossman syndrome, increased levels of complement components C1QA and C1QB, along with lens structural proteins such as gamma-crystallin (CRYGS) and beta-crystallin (CRYBB2) were identified.

### Vogt-Koyanagi-Harada disease and Behcet’s uveitis

Zhang et al. [41] compared the proteome of AH-derived exosomes in VKH disease and Behcet’s uveitis with control samples obtained before cataract surgery. The AH of eyes with VKH and Behcet’s uveitis were all obtained at a time of clinically inactive disease status. The study identified 65 differentially expressed proteins in VKH (40 upregulated, 25 downregulated) and 40 in Behcet’s uveitis (38 upregulated, 2 downregulated). Proteins involved in complement-related pathways were dominant in the pathway analyses.

### Cytomegalovirus-hypertensive anterior uveitis

Choi et al. compared the AH of eyes with CMV-HAU to that of eyes with senile cataract that was used as control group. They identified 65 differentially expressed proteins, 50 of which were upregulated and 15 downregulated [38]. Regulated proteins in cytomegalovirus-associated uveitis were predominantly involved in complement activation, circulating immunoglobulin-mediated humoral responses, proteolysis, and platelet degranulation, indicating abnormal regulation of complement-mediated inflammation and immune responses. Additionally, the study showed that CD14 was significantly elevated (~ 50-fold) in the AH of CMV-HAU suggesting an activation of the innate immune system. The humoral immune response mediated by circulating immunoglobulin was the second most enriched pathway in CMV-HAU AH.

### Idiopathic intermediate uveitis

Sepah et al. [36] compared the proteome of the VH in eyes with idiopathic intermediate uveitis to those with idiopathic macular hole.

Enriched pathways included complement system, atherosclerosis signaling, coagulation system, clathrin-mediated endocytosis, intrinsic prothrombin activation, glycolysis, and IL-12 signaling in macrophages.

### Juvenile idiopathic arthritis (JIA) uveitis and silent chronic anterior uveitis

Ayuso et al. [35] performed a proteomic analysis of the AH from four distinct groups: JIA-associated uveitis, silent chronic anterior uveitis, eyes with other types of uveitis, and control eyes from either cataract or congenital glaucoma. Overall, JIA and silent chronic anterior uveitis samples displayed similar protein profiles,including an upregulation of transthyretin in both subtypes.

### Risk of bias within individual studies

Table 4 provides a detailed overview of the risk of bias for each study. Ayuso et al. [35], Komatsu et al. [37], Choi et al. [38], Tao et al. [39], and Zhang et al. [41] were all evaluated as having low risk of bias in most categories, including source definition, eligibility criteria, time period and quality assurance. However, none of these studies explicitly mentioned consecutive recruitment. This leaves it unclear whether every eligible participant who met the study criteria during the recruitment period was included. Furthermore, no study indicated that any patients were excluded after initial inclusion. As there was no explicit statement confirming that no later exclusions took place, this introduces some uncertainty regarding potential selection biases. Sepah et al. [36], being a case study, naturally did not address consecutive recruitment and time period, which is typical for this type of study design. Sepah et al. [36] maintained clear source definition, eligibility criteria, and quality assurance, like the other studies. However, it also did not explicitly mention the absence of later exclusions.

**Table 4:** Risk of bias within individual studies using the Agency for Healthcare Research and Quality checklist for Cross-Sectional Studies

Reference	Source Definition	Eligibility criteria	Time period	Consecutive recruitment	Quality assurance	Exclusion explanation
Ayuso et al. [35]	Yes	Yes	Yes	Unclear	Yes	Unclear/No exclusions
Sepah et al. [36]	Yes	Yes	No	No	Yes	Unclear/No exclusions
Komatsu et al. [37]	Yes	Yes	Yes	Unclear	Yes	Unclear/No exclusions
Choi et al. [38]	Yes	Yes	Yes	Unclear	Yes	Unclear/No exclusions
Tao et al. [39]	Yes	Yes	Yes	Unclear	Yes	Unclear/No exclusions
Zhang et al. [41]	Yes	Yes	Yes	Unclear	Yes	Unclear/No exclusions

**The assessment of studies involves evaluating the studies with Yes/No/Unclear responses based on the following criteria from the Agency for Healthcare Research and Quality checklist:**

Source Definition: Identifies the source of the information

Eligibility Criteria: Specifies the inclusion and exclusion criteria or references previous publications

Time Period: States the time frame used for participant identification

Consecutive Recruitment: Indicates if the participants were consecutively recruited

Quality Assurance: Details any quality assurance assessments conducted

Exclusion Explanation: Clarifies any patient exclusions from the analysis

## Discussion

This systematic review aimed to summarize findings from proteomic studies that compared uveitis patients to controls. The results indicate significant variability in differentially expressed proteins across studies. This reflects the diverse etiology and multifactorial pathophysiology of various uveitis types. Each study identified numerous differentially expressed proteins, highlighting the complex interplay of many proteins underlying uveitis. Despite these differences, in most studies elevated levels of complement, coagulation and inflammatory factors to varying degrees were observed.

The similar clinical presentations of various uveitis types may conceal the different underlying pathogeneses. This could lead to initial treatment failures and delays in effective therapy, which might result in vision loss. The intraocular proteome presents an attractive gateway to unravel the pathophysiological pathways underlying different uveitis entities.

### Consistently upregulated proteins

One of the significant findings across multiple studies was the consistent upregulation of complement proteins such as C1q subcomponents. Specifically, complement C1q subcomponent subunit B and complement C1q subcomponent subunit C were consistently upregulated in five studies. Complement activation plays a critical role in inflammation and immune response. The complement system is a vital part of both the innate and adaptive immune systems. It enhances the effectiveness of antibodies and phagocytes [42]. C1q is historically known to initiate the classical complement pathway. This leads to opsonization of pathogens and promotion of phagocytosis. C1q is crucial in host defense as it acts as a pattern recognition molecule, capable of identifying both self and non-self ligands. It signals immediate and long-term protective functions [43, 44]. Research suggests that C1q could help Behcet’s uveitis patients in combating pathogens, which are identified as crucial triggers in the progression of Behcet’s uveitis [45, 46].

Three proteins − Alpha-2-HS-glycoprotein, Apolipoprotein A-I, and alpha-1-antichymotrypsin − were upregulated in four studies. Alpha-2-HS-glycoprotein (fetuin-A) is involved in various physiological processes, including inflammation and immune regulation. It acts as an inhibitor of ectopic calcification and has anti-inflammatory properties [47]. Apolipoprotein A-I plays a crucial role in lipid metabolism and has anti-inflammatory properties. It is the main protein component of high-density lipoprotein in plasma, involved in cholesterol efflux and reverse cholesterol transport, which can modulate immune responses and inflammation [48]. In an animal study, researchers discovered that administering Apolipoprotein A-I alleviates autoimmune uveitis by regulating the balance between harmful and regulatory T cells suggesting a therapeutic potential [49]. Alpha-1-antichymotrypsin is a serine protease inhibitor that protects tissues from enzymes of inflammatory cells, especially elastase and cathepsin G, thereby regulating inflammatory responses [50]. Alpha-1-antichymotrypsin is known to play a role in various ophthalmologic conditions. It has anti-inflammatory, anti-angiogenic, antioxidant, and antifibrotic effects, including its protective effects on Müller cells against oxidative stress [51].

Ceruloplasmin was upregulated in four studies but downregulated in the study by Tao et al. [39]. This indicates that ceruloplasmin may have a different role in Fuchs uveitis, studied by Tao et al. [39] than other uveitis types. Ceruloplasmin is a ferroxidase enzyme that plays a role in iron metabolism. It oxidizes ferrous iron to ferric iron, which facilitates its incorporation into transferrin. It also has antioxidant properties, protecting tissues from oxidative damage [52]. Previous reports have suggested a protective role of ceruloplasmin in neurodegenerative diseases. However, it is still uncertain whether iron accumulation is a cause or a consequence of neurodegeneration [53]. The variability in ceruloplasmin expression in uveitis is not yet fully understood, and further research is needed to clarify its significance.

Similarly, three proteins—gelsolin (AGEL), kininogen-1 (alpha-2-thiol proteinase inhibitor), and alpha-1-antitrypsin—were upregulated in three studies, but downregulated in the study by Tao et al. [39]. Gelsolin is an actin-binding protein that regulates actin filament assembly and disassembly, playing a role in cell motility, shape, and apoptosis. Gelsolin was found to be increased in other pathological ophthalmic conditions as well: It was increased in several proteomics studies of the VH of eyes with diabetic macular oedema [54–56] and in the tear fluid of eyes with dry eye disease [57]. Kininogen-1 is involved in the kinin-kallikrein system, which regulates blood pressure, coagulation, and inflammation. It acts as a precursor for kinins, which are strong vasodilators and modulators of inflammatory responses. In both systemic circulation and ocular tissues, kinin-kallikrein system-derived peptides can strengthen signaling pathways mediated by pro-inflammatory and angiogenic factors. These include vascular endothelial growth factor, cytokines and chemokines. Kininogen-1 was linked to the inflammatory and proliferative changes observed in various ocular diseases, including diabetic macular edema, diabetic retinopathy, glaucoma, uveitis, and age-related macular degeneration [58–62]. The kinin-kallikrein system may increase retinal permeability and edema, as seen in patients with diabetic retinopathy, where elevated levels of carbonic anhydrase raise intraocular pH. This activates plasma kallikrein to generate bradykinin, a bioactive peptide derived from its precursor, kininogen [63]. Alpha-1-antitrypsin, like alpha-1-antichymotrypsin, is a serine protease inhibitor that plays a critical role in protecting tissues from inflammatory cell enzymes, particularly neutrophil elastase. By modulating inflammatory responses and limiting tissue damage, alpha-1-antitrypsin contributes to maintaining tissue integrity [64]. Although primarily created in the liver, AAT production has also been detected in corneal epithelial cells [65, 66]. Similar to alpha-1-antichymotrypsin, alpha-1-antitrypsin has been implicated in various ocular diseases, including keratitis, diabetic retinopathy, age-related macular degeneration, glaucoma, cataracts, dry eye disease, keratoconus, uveitis and pterygium [67].

A hereditary deficiency in alpha-1-antitrypsin, known as α−1-antitrypsin deficiency, is an autosomal disorder affecting the SerpinA1 gene. It has been linked to conditions such as ocular allergy and uveitis [68].

### Perspectives on the individual studies

Komatsu et al. [37] found upregulated NGAL and upregulated JAM-B in ocular sarcoidosis compared to vitreoretinal lymphoma and controls with macular holes and epiretinal membrane. NGAL, also known as lipocalin-2, is an adipokine involved in metabolic homeostasis, apoptosis, infection, immune response, and inflammation [69–72]. Increased NGAL expression has been linked to C. acnes and various inflammatory diseases. Previous studies have shown high NGAL levels in the VH in conditions like rhegmatogenous retinal detachment and proliferative diabetic retinopathy (PDR) [73–77]. Komatsu et al. [37] suggested that although NGAL levels in ocular sarcoidosis are lower than in PDR, the distinct clinical presentations of these diseases reduce the likelihood of misdiagnosis. JAM-B, a member of the immunoglobulin superfamily, is primarily expressed in vascular endothelial cells and supports leukocyte recruitment [78]. It is more extensively expressed in tissues with chronic inflammatory diseases such as asthma and autoimmune hepatitis [79]. The authors proposed that both JAM-B and NGAL should be considered as potential indicators for distinguishing ocular sarcoidosis, because NGAL is also elevated in proliferative diabetic retinopathy (PDR) [37].

Tao et al. [39] observed increased activity of the hypoxia pathway, indicating it could play a role in Fuchs uveitis syndrome pathogenesis. It also suggested that HIF-1 may be of importance. They hypothesized a complex interplay between hypoxia and inflammation in Fuchs uveitis syndrome [39]. This aligns with previous research linking hypoxia, infection and inflammation. Hypoxia may induce inflammation. Chronic inflammation, in turn, increases oxygen consumption, maintaining hypoxia [80]. Intracellular pathogens could worsen this hypoxic state. Infectious agents such as herpes simplex virus, rubella virus, cytomegalovirus, and ocular toxoplasmosis have all been reported in association with Fuchs uveitis syndrome [81–83]. The release of β2-microglobulin (B2M) from tissues may serve to suppress inflammation and inhibit microbial proliferation [84–86]. This could explain the elevated B2M levels found in Fuchs uveitis syndrome patients in the study [39]. Hypoxia-inducible factor 1 (HIF-1), a key regulator of cellular responses to hypoxia, has been linked to several immune disorders. Recent studies suggest that certain antioxidant and anti-inflammatory agents can reduce the severity of anterior uveitis by modulating HIF-1α and TNF-α signaling pathways [87, 88].

As stated in the results, Zhang et al. [41] found significant increases in the expression of ceruloplasmin and C1QB in Behcet’s uveitis and VKH. Previous research indicates that fibroblasts, non-pigmented epithelial cells, and lens epithelial cells may also produce exosomes carrying ceruloplasmin, which functions as an acute-phase reactant during inflammation [89]. Macrophages play a critical role in maintaining immune homeostasis within the eye [90]. In a recent study, C1Q-high monocytes were found to enhance phagocytosis and secrete pro-inflammatory cytokines in Behçet’s disease [91]. In various studies, elevated ceruloplasmin expression has been associated with various inflammatory diseases, including ankylosing spondylitis, rheumatoid arthritis, and Behçet’s disease [92–94]. As the AH was collected from eyes with clinically inactive disease, these findings suggest the presence of subclinical inflammation in the anterior segment, continuing even after clinical manifestations have ended [41]. This supports a broader therapeutic perspective, consistent with previous studies [95, 96], that extended treatment is often necessary to achieve lasting control of intraocular inflammation in both conditions [40, 41].

Choi et al. found that complement activation was the most prominent pathway in the AH of CMV-HAU patients [38]. Previous research has shown that the complement cascade generates mediators that damage cells directly and activate innate immune cells [97]. Aberrations in the complement system have been implicated in both glaucoma and uveitis in other studies [98, 99].

In Choi et al., in the proteomic analysis of CMV-HAU AH, vasorin levels were significantly decreased [38]. Vasorin is known to be an anti-TGF- β glycoprotein. Previous research has shown that the ocular microenvironment contains immunosuppressive mediators like TGF-β, macrophage migration inhibitory factor, and α-MSH. These influence immune responses and intraocular pressure (IOP) [100–102]. CMV infection induces TGF-β secretion, modifying immune reactions to favor viral replication [103]. The decreased vasorin in Choi et al. indicates elevated TGF-β activity contributing to CMV-HAU’s clinical characteristics of mild inflammation but high IOP [38]. Lastly, Choi et al. found a ninefold increase in myocilin levels in CMV-HAU AH [38]. Myocilin, highly expressed in the trabecular meshwork, can be stimulated by dexamethasone, retinoic acid, and TGF-β [104–106]. While the link between myocilin overexpression and IOP elevation is unclear, steroid and anti-glaucomatous eyedrops used during the study’s follow-up period may have contributed to increased myocilin levels in CMV-HAU patients [38]. CMV infection is a notable cause of hypertensive anterior uveitis in immunocompetent patients [81, 107]. In cases of anterior uveitis, the associated ocular hypertension often results in glaucomatous damage, occurring in about 20–30% of cases [108, 109]. Clinically, CMV-HAU presents not only with recurrent uveitis but also with challenging IOP management. This leads to a significantly higher risk of glaucoma surgery compared to hypertensive anterior uveitis cases without CMV [81, 107, 110].

Sepah et al. found fibronectin—a protein involved in organizing extracellular matrix components and collagen—to be significantly increased. They suggested that this increase may contribute to the development of the characteristic snowballs in intermediate uveitis [36]. This aligns with previous research showing that in intermediate uveitis patients, "snowballs" often form within the VH, mainly composed of glial elements, Müller cells, organized collagen, and inflammatory cells [111]. Furthermore, Sepah et al. found the complement cascade one of the most represented pathways. The complement cascade has been previously linked to the development of uveitis [36]. Sepah et al. has identified increased levels of complement cascade components such as C1, C1R, C1S, and C1QS, along with 22 IL-12 signaling mediators, in samples from patients with intermediate uveitis [36]. It is well known that IL-12—a factor produced by neutrophils, dendritic cells, and macrophages in response to antigens – promotes the differentiation of naïve CD4 T-cells into Th1-effector cells [112, 113]. In previous studies, the roles of Th1 and Th17 cells in experimental autoimmune uveitis have been thoroughly investigated [114–116]. Findings from previous studies indicate that IL-12 also has the ability to inhibit the development of autoimmune diseases by inducing the expression of interferon gamma (IFN-γ). Thus, it highlights the involvement of both innate and adaptive immune systems in the pathogenesis of intermediate uveitis [117, 118]. Moreover, Sepah et al. found IL-23 to be a critical upstream regulator [36]. IL-23 signaling was previously linked to increased levels of myeloid-specific proteins S100A8 and A9 [119]. Both of these were detected in the proteomic analysis of intermediate uveitis samples [36]. S100A8, known to promote inflammatory cell migration and infiltration, has previously been associated with acute anterior uveitis and the recurrence of experimental autoimmune uveitis in animal models [120, 121]. Upregulation of IL-23 induces the differentiation of naïve CD4 + T cells into IL-17 helper T cells (TH17/THIL-17). IL-17 enhances T cell priming and stimulates fibroblasts, endothelial cells, macrophages, and epithelial cells to produce pro-inflammatory mediators, such as IL-1, IL-6, TNF alpha, NOS-2, and metalloproteases, leading to inflammation [122]. The IL-23/IL-17 pathway is critical in several autoimmune diseases, including psoriatic skin inflammation, inflammatory bowel disease, experimental autoimmune encephalitis and autoimmune myocarditis [123, 124]. High IL-23 expression in intermediate uveitis suggests its role in the disease’s pathology, indicating that targeting IL-23 signaling could be a viable therapeutic strategy for intermediate uveitis patients [36].

Ayuso et al. [35] found transthyretin to be upregulated in JIA uveitis and silent chronic anterior uveitis. Transthyretin is primarily known for transporting thyroid hormones and retinol-binding protein (RBP), but recent evidence indicates its involvement in various biological processes [125–127]. Transthyretin is secreted by retinal pigment epithelium cells, choroid tissues, and retinal microvascular endothelial cells. It is found in nearly all ocular tissues, including the AH [127–129]. In ocular diseases, it plays different roles. Transthyretin levels are decreased in the AH of diabetic retinopathy patients compared to controls and have been reported to suppress neovascularization [130]. Ayuso et al. suggested that transthyretin may play a role in the pathogenesis of JIA-associated uveitis. However, they also hypothesized that transthyretin expression might be a consequence of this uveitis, serving as a marker for silent chronic non-infectious anterior uveitis. Further investigation is needed to clarify this [35].

### Limitations

The included studies in this review were limited by the number of subjects, sample volumes, and protein concentrations. Small sample sizes inherently reduce the statistical power of studies, increasing the chance of type II errors, where genuine differences or associations could be overlooked. For example, in the study by Sepah et al., the study group consisted of VH samples from four eyes of three patients with intermediate uveitis, while the control group consisted of VH samples from four eyes of four patients with macular holes [36]. However, uveitis comprises a large variety of inflammatory entities, which can make it difficult to achieve large sample sizes. An important limitation of our study is that each disease-specific analysis relied on a single study.

The control groups in the reviewed studies varied in their inclusion criteria. For example, Ayuso et al. included six patients with congenital glaucoma in their control group [35]. Research indicates that eyes affected by glaucoma exhibit a distinct proteomic profile compared to healthy controls [40, 131–133]. The other studies in the review used control groups consisting of eyes with macular holes, epiretinal membrane, or cataract that are widely accepted as controls [28]. Due to the heterogeneity across the control groups, it was not feasible to conduct a meta-analysis. While a number of proteome changes were identified across the uveitis studies, it is important to keep in mind that uveitis encompasses a very heterogenous disease group. Further studies of the same uveitis subtypes may help validate and establish the specific proteomes related to uveitis subtypes.

Four out of the six studies had a difference of more than 10 years between the mean age of the control group and the mean age of the study group [36, 38, 39, 41]. Only the studies by Komatsu et al. and Ayuso et al. had a less than 10-year difference in mean age between the study and control groups [35, 37]. This could influence the proteomic profile given its known variability with age [134, 135]. However, Sepah et al. argued that the differentially expressed proteins primarily belonged to inflammatory pathways, which are not typically highly expressed in healthy individuals, regardless of age. This suggests that the observed differentially expressed proteins were unlikely to be age-related [36].

Four studies collected samples between 2016 and 2024 [37–39, 41]. One study did not mention the timeframe of sample collection [36], and another study collected samples from 2004 to 2010 [35]. The inclusion and exclusion criteria varied among the studies but were overall sufficiently described.

Generally, gender matching was not achieved in these studies. This was likely due to the limited availability of study participants and the fact that certain types of uveitis occur more often in one gender.

In five studies, a subgroup of patients in the study groups had received systemic or topical corticosteroid treatments, systemic immunosuppressive treatments, or other eyedrop medication to some extent. Komatsu et al. included patients with ocular sarcoidosis or vitreoretinal lymphoma who had not received corticosteroids for one month before vitrectomy, although prior steroid treatments were common [37]. Zhang et al. reported that patients took systemic corticosteroids and immunosuppressants before sample collection [41]. Tao et al. indicated that patients underwent topical corticosteroid therapy before sample collection [39]. In the study by Sepah et al., two out of three patients in the study group received corticosteroid treatment before VH samples were taken [36]. In Choi et al. the study group had used anti-glaucoma eyedrops, which were not further specified, before sample collection [38]. Ayuso et al. did not mention pre-sample collection treatments [35]. These treatments may have affected the VH or AH samples. Ideally, studies should analyse treatment-naive eyes to obtain a clearer understanding of the disease pathology. However, analyzing VH samples from patients with a recent history of corticosteroid treatment is common in clinical practice, as eyes with acute inflammation typically receive steroid treatments before undergoing vitrectomy. Nonetheless, sampling from non-treatment-naive eyes or those with inactive uveitis may not fully reflect the pathogenesis of active uveitis.

Another limitation is the pooling of samples. This was done in the studies by Zhang et al. [41] and Tao et al. [39]. Zhang et al. [41] noted that this was due to the relatively large volume of AH required for exosome isolation and proteomic analysis. Mixed AH samples from patients were used in both studies [39, 41]. Because of this, the studies could not reflect the results of each individual patient. In this context, small sample volumes and low protein concentrations might have been limitations in all studies. Often, the entire material may be used for proteomic analysis without leaving options for validation with ELISA or Western blotting.

### Technical aspects/methodological variability

The variability in proteomic technologies used across studies also poses a challenge. Five out of six studies used LC–MS/MS, whereas Ayuso et al. [35] used Seldi-ToF MS. Advanced techniques like LC–MS/MS offer high sensitivity and specificity but are prone to technical biases such as ion suppression and matrix effects [136–138]. In LC–MS/MS, ion interference can negatively impact specificity and accuracy [139]. Older methods like SELDI-ToF MS may lack the resolution and accuracy needed for reliable protein identification and quantification.

Validation of proteomic findings is crucial for establishing their clinical relevance. While several studies used ELISA, HR-MRM, or Western blotting to validate their findings, the extent of validation varied. For instance, Komatsu et al. and Ayuso et al. used ELISA for selected proteins [35, 37], whereas Tao et al. used HR-MRM and Western blotting for some of their identified proteins, providing a better validation for those specific proteins [39]. This variation in validation rigor can impact the confidence in the reported findings.

As all proteomic technologies have their advantages and disadvantages, it would add further strength to the results of the studies in this review to have a systematic approach to validation. This involves validating more proteins identified in the discovery phase, using multiple independent methods, and replicating findings across different cohorts. Multi-platform proteomic analysis is a possibility to get reliable results. For example, Dammer et al. used three proteomic platforms to analyze body fluid: SomaScan, Olink proximity extension assay and tandem mass tag-based mass spectrometry [140]. Key proteins found cross-platform have increased confidence/reliability [141, 142]. Additionally, longitudinal studies that track changes in protein expression over time can provide insights into the dynamics of uveitis and its response to treatment.

Our review encompassed six studies published between 2013 and 2024, reflecting over a decade of advancements in proteomic techniques. Significant improvements in sample preparation, liquid chromatography, and mass spectrometry have been made during this period. Advancements in proteomics techniques enable the identification of more proteins with higher confidence, resulting in more comprehensive coverage of the uveitis proteome. This provides deeper insights into the mechanisms and underlying pathophysiology of the disease. Personalized medicine becomes possible with these advancements. This allows for the discovery of proteins that not only aid in diagnosing specific types of uveitis but also reveal highly effective treatment options, thereby preventing vision loss.

## Conclusion

Despite significant differences in the pathogenesis among various types of uveitis, there was a notable overlap in the analyses, with several studies identifying changes in the same proteins. Notably, complement C1q subcomponent subunit B and complement C1q subcomponent subunit C were consistently upregulated in five of six studies. Additionally, three proteins—alpha-2-HS-glycoprotein, apolipoprotein A-I, and alpha-1-antichymotrypsin—were upregulated in four studies. These consistent proteomics findings suggest the activation of common pathways in various types of intraocular inflammation. Longitudinal studies, tracking changes in protein expression over time and correlating significantly regulated proteins with clinical parameters, may enhance our understanding of treatment targets. Additionally, future research may focus on developing standardized protocols and on distinguishing between different uveitis types. This may hold the potential to further enhance, accelerate, and personalize the diagnosis and treatment of uveitis.

## Supplementary Information

Additional file1

Additional file2

Additional file3

## Data Availability

All data generated or analyzed during this study are included in this published article.
