# Supplementary material for: Proteomics analysis of aqueous and vitreous humor in uveitis: a systematic literature review
Source: Clin Proteomics. 2025 Dec 16;23:3. doi: 10.1186/s12014-025-09564-2 (PMC12821945; doi:10.1186/s12014-025-09564-2)
Supplement: Supplementary file 1 — Additional file1 [file 12014_2025_9564_MOESM1_ESM.docx]

**Supplementary file 1.** Details of the literature search.

Search phrases and results from databases (Pubmed, Cochrane, Embase, Web of Science) on 26^th^ of January 2024.

**Pubmed**


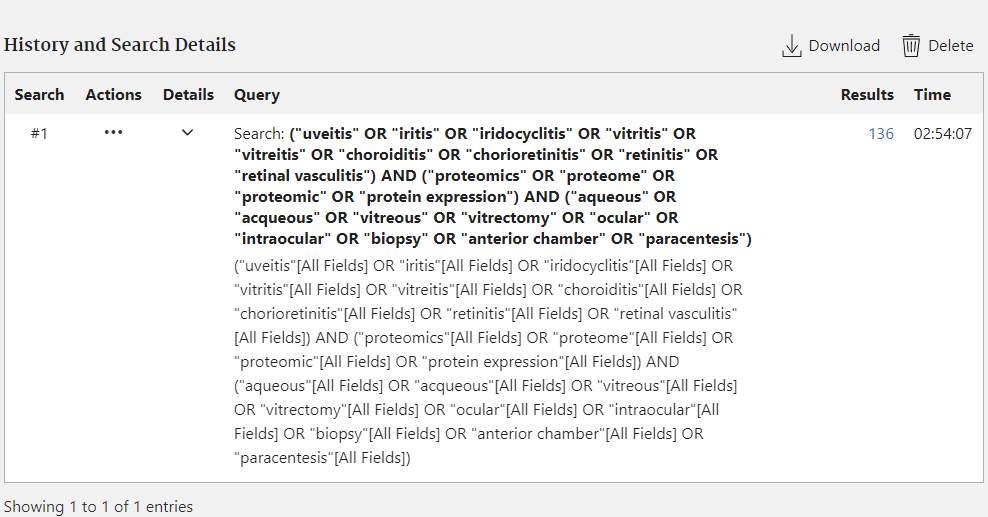
Link to search in Pubmed: [136](https://pubmed.ncbi.nlm.nih.gov/?term=%28%22uveitis%22+OR+%22iritis%22+OR+%22iridocyclitis%22+OR+%22vitritis%22+OR+%22vitreitis%22+OR+%22choroiditis%22+OR+%22chorioretinitis%22+OR+%22retinitis%22+OR+%22retinal+vasculitis%22%29+AND+%28%22proteomics%22+OR+%22proteome%22+OR+%22proteomic%22+OR+%22protein+expression%22%29+AND+%28%22aqueous%22+OR+%22acqueous%22+OR+%22vitreous%22+OR+%22vitrectomy%22+OR+%22ocular%22+OR+%22intraocular%22+OR+%22biopsy%22+OR+%22anterior+chamber%22+OR+%22paracentesis%22%29&sort=)

**Cochrane Library**

**39**Trials matching **("uveitis" OR "iritis" OR "iridocyclitis" OR "vitritis" OR "vitreitis" OR "choroiditis" OR "chorioretinitis" OR "retinitis" OR "retinal vasculitis") AND ("proteomics" OR "proteome" OR "proteomic" OR "protein expression") AND ("acueous" OR "aqueous" OR "acqueous" OR "vitreous" OR "vitrectomy" OR "ocular" OR "intraocular" OR "biopsy" OR "anterior chamber" OR "paracentesis") in Title Abstract Keyword - (Word variations have been searched)**

[Cochrane Central Register of Controlled Trials](https://www.cochranelibrary.com/)

Issue 1 of 12, January 2024

**Embase**

**Link:**
[Click to run search](https://ep.fjernadgang.kb.dk/login?url=http://ovidsp.ovid.com/ovidweb.cgi?T=JS&NEWS=N&PAGE=main&SHAREDSEARCHID=1ToDPv2la4iN6yrRnY2zKcTDMhmGIP6RP16eD5CwITL6ZElxvTYxqRnyP9bWn4Wfk)
The above Jumpstart will only work for users who have access to this specific database.

| **#** | **Query** | **Results from 26 Jan 2024** |
| --- | --- | --- |
| 1 | (uveitis or iritis or iridocyclitis or vitritis or vitreitis or choroiditis or chorioretinitis or retinitis or "retinal vasculitis").ti,ab,kw. | 54,758 |
| 2 | (proteomics or proteomic or proteome or "protein expression").ti,ab,kw. | 399,183 |
| 3 | (acueous or acqueous or aqueous or vitreous or vitrectomy or ocular or intraocular or biopsy or "anterior chamber" or paracentesis).ti,ab,kw. | 1,154,774 |
| 4 | 1 and 2 and 3 | 140 |

**Web of Science**

Query Link: <https://www.webofscience.com/wos/alldb/summary/801abb4d-a462-4c32-a125-af2d8fbf7e0d-cdd79d5d/relevance/1>

# Web of Science Search Strategy (v0.1)

# Database: All Databases

# Entitlements:

- WOS: 1900 to 2024

- BIOSIS: 1969 to 2024

- CCC: 1998 to 2024

- DRCI: 1900 to 2024

- DIIDW: 1969 to 2024

- GRANTS: 1953 to 2024

- KJD: 1980 to 2024

- MEDLINE: 1950 to 2024

- PPRN: 1991 to 2024

- PQDT: 1637 to 2024

- SCIELO: 2002 to 2024

- ZOOREC: 1976 to 2024

# Searches:

1: TS=( "Uveitis" OR "iritis" OR "iridocyclitis" OR "vitritis" OR "vitreitis" OR "choroiditis" OR "chorioretinitis" OR "retinitis" OR "retinal vasculitis") AND TS=("proteomics" OR "proteomic" OR "proteome" OR "protein expression") AND TS=("acueous" OR "acqueous" OR "aqueous" OR "vitreous" OR "vitrectomy" OR "ocular" OR "intraocular" OR "biopsy" OR "anterior chamber" OR "paracentesis") and Preprint Citation Index (Exclude – Database) Date Run: Fri Jan 26 2024 08:02:10 GMT+0100 (Central European Standard Time) Results: 280
