# Supplementary material for: Proteomics analysis of aqueous and vitreous humor in uveitis: a systematic literature review
Source: Clin Proteomics. 2025 Dec 16;23:3. doi: 10.1186/s12014-025-09564-2 (PMC12821945; doi:10.1186/s12014-025-09564-2)
Supplement: Supplementary file 3 — Additional file3 [file 12014_2025_9564_MOESM3_ESM.docx]

***Ocular Sarcoidosis***

Komatsu et al. (2022) performed a proteome analysis on the vitreous humor of eyes affected by ocular sarcoidosis and control samples consisting of vitreous samples from eyes with epiretinal membrane or macular hole. To confirm selected proteins identified by mass spectrometry and to compare with other types of uveitis, ELISA was performed on samples from eyes with ocular sarcoidosis, vitreoretinal lymphoma (VRL), and Behçet’s disease. However, no proteome analysis was carried out on the vitreous humor of eyes with Behçet’s disease. The primary comparison focused on the proteome of the vitreous humor from eyes with ocular sarcoidosis and those with epiretinal membrane or macular hole (Komatsu et al., 2022).

The study identified 263 upregulated proteins and 27 downregulated proteins. Using the Reactome pathway database, they analyzed the top 10 pathways associated with differentially expressed proteins in ocular sarcoidosis versus controls. Among these pathways, neutrophil granulation, associated with the innate immune system, was the most significantly upregulated in ocular sarcoidosis. Notably, eight out of the top ten pathways were part of the broader immune system pathway. For downregulated pathways in ocular sarcoidosis compared to controls, collagen chain trimerization was the most significantly downregulated (Komatsu et al., 2022).

Neutrophil gelatinase-associated lipocalin (NGAL) and junctional adhesion molecule B (JAM-B) were selected as candidate proteins for distinguishing ocular sarcoidosis from similar diseases. ELISA assays on samples from eyes with ocular sarcoidosis, vitreoretinal lymphoma, and Behçet’s disease showed that NGAL was significantly upregulated in ocular sarcoidosis compared to vitreoretinal lymphoma, controls, and Behçet’s disease. Similarly, JAM-B concentrations were increased significantly in ocular sarcoidosis compared to vitreoretinal lymphoma and controls (Komatsu et al., 2022).

***Fuchs Uveitis Syndrome***

**Tao et al. (2024)** compared the proteome of the AH from eyes with Fuchs uveitis syndrome to the proteomes of age-related cataract and Posner-Schlossman syndrome. Notably, the proteome of the AH from eyes with Posner-Schlossman syndrome was not compared to that from eyes with age-related cataract. In the primary comparison of the proteome of Fuchs uveitis and controls, Tao et al. (2024) identified 174 differentially expressed proteins, with 78 upregulated and 96 downregulated. Upregulated proteins included beta-2-microglobulin (B2M), alpha 1-acid glycoprotein 1 (ORM1), complement C1q subcomponent subunit A (C1QA), complement C1s subcomponent (C1S), complement C1q subcomponent subunits B (C1QB) and C (C1QC), and matrix metalloproteinase-9 (MMP9). On the other hand, testican-1 (SPOCK1) and reelin (RELN), related to extracellular matrix formation, were significantly downregulated (Tao et al., 2024).

Pathway analysis with Gene Ontology, Kyoto Encyclopedia of Genes and Genomes, and Pathway Responsive GENes indicated that the hypoxia-inducible factor-1 (HIF-1) signaling pathway, acute inflammatory response, humoral immune response, response to bacterium, viral infection, FCyR-mediated phagocytosis and complement and coagulation cascades are significant in mediating Fuchs uveitis syndrome (Tao et al., 2024).

In the comparative analysis of the differential proteomic profiles between Fuchs uveitis syndrome and Posner-Schlossman syndrome, 50 upregulated and 21 downregulated differentially expressed proteins were identified. Notably, complement components C1QA and C1QB, along with lens structural proteins such as gamma-crystallin (CRYGS) and beta-crystallin (CRYBB2), were significantly upregulated in Fuchs uveitis syndrome. In contrast, SPARC, a protein known for its role in preventing cataract formation, was significantly downregulated. The two upregulated proteins, C1QB and secretogranin-1 (CHGB), were validated by HR-MRM (Tao et al., 2024).

***Vogt-Koyanagi-Harada disease and Behcet’s uveitis***

**Zhang et al. (2024)** compared the proteome of AH-derived exosomes in VKH disease and Behcet’s uveitis with control samples obtained before cataract surgery. The AH of eyes with VKH and Behcet’s uveitis were all obtained at a time of clinically inactive disease status. This was also indicated by the absence of inflammatory cells in the AH. The study identified 65 differentially expressed proteins in VKH (40 upregulated, 25 downregulated) and 40 in Behcet’s uveitis (38 upregulated, 2 downregulated). Proteins involved in complement-related pathways were dominant in the pathway analyses. Significant increases in the expression of ceruloplasmin and C1QB were observed in both groups, Behcet’s uveitis and VKH, so they were selected as key exosomal proteins for further study. Western blotting confirmed the proteomic findings in another set of AH exosomes from these three groups, consistently showing increased expression of these two proteins in both VKH and Behcet’s uveitis. The differentially expressed proteins of AH exosomes were compared with the differentially expressed proteins of AH. The differentially expressed proteins in the AH from the VKH group did not include C1QB, suggesting the presence of concentrated C1QB only within the exosomes. The authors concluded that the two key exosomal proteins − complement component C1QB and ceruloplasmin − were potentially involved in the development of both diseases. The authors hypothesized that monocyte-derived macrophages in the iris, ciliary body, and trabecular meshwork were the primary source of exosomes containing C1QB (Zhang et al., 2024).

***Cytomegalovirus-hypertensive anterior uveitis***

**Choi et al. (2022)** compared the AH of eyes with CMV-HAU to that of eyes with senile cataract, the latter being the control group. They identified 65 differentially expressed proteins, 50 of which were upregulated and 15 downregulated (Choi et al., 2022). complement activation, circulating immunoglobulin-mediated humoral responses, proteolysis, and platelet degranulation. Furthermore, STRING analysis revealed the presence of genes enriched for roles in phospholipid efflux and complement activation. These findings were reported to indicate abnormal regulation of complement-mediated inflammation and immune responses. This was suggested to contribute to ocular hypertension and glaucoma in CMV-HAU cases (Choi et al., 2022). The study reported that complement activation was the most prominent pathway in the AH of CMV-HAU patients (Choi et al., 2022). Additionally, the study showed that CD14 was significantly elevated (~50-fold) in the AH of CMV-HAU suggesting an activation of the innate immune system. The humoral immune response mediated by circulating immunoglobulin was the second most enriched pathway in CMV-HAU AH (Choi et al., 2022).

Vasorin levels in the AH of eyes with CMV-HAU were significantly decreased. Vasorin is known to be an anti-TGF- β glycoprotein (Ikeda et al., 2004; Malapeira et al., 2011). In contrast, myocilin levels were a 9-fold increased (Choi et al., 2022).

***Idiopathic intermediate uveitis***

**Sepah et al. (2020)** compared the proteome of the vitreous humor in eyes with idiopathic intermediate uveitis to those with idiopathic macular hole. They identified 233 differentially expressed proteins, with 103 upregulated and 130 downregulated. In intermediate uveitis, significantly upregulated proteins included latent transforming growth factor beta-binding protein 2 (LTBP2), retinoic acid receptor responder protein 2 (RARR2), ribonuclease 1 (RNAS1), peptidoglycan recognition protein 2 (PGRP2), ceruloplasmin (CERU), biotinidase (BTD), afamin (AFAM), anti-thrombin III (ANT3), fibronectin (FN), transthyretin, cystatin-3 (CYTC), and alpha-1B glycoprotein (A1BG). Conversely, downregulated proteins included cystatin-S (CST4), glutathione synthetase (GSS), calsyntenin-3 (CLSTN3), tryptophanyl-tRNA synthetase (WARS), prolyl 4-hydroxylase beta polypeptide (P4HB), clusterin-like 1 (CLUL1), and aspartylglucosaminidase (AGA) (Sepah et al., 2020).

Sepah et al. (2020) used Ingenuity Pathway Analysis where the most represented pathways were liver x receptor/retinoid x receptor activation, acute phase response, farsenoid x receptor/retinoid x receptor activation, complement system, atherosclerosis signaling, coagulation system, clathrin-mediated endocytosis, intrinsic prothrombin activation, glycolysis and IL-12 signaling in macrophages (Sepah et al., 2020).

***Juvenile idiopathic arthritis (JIA) uveitis and silent chronic anterior uveitis***

**Ayuso et al. (2013)** performed a proteomic analysis of the AH from four distinct groups: Eyes with JIA uveitis, eyes with silent chronic anterior uveitis, eyes with other types of uveitis, and control eyes (from patients with cataracts and congenital glaucoma). The study involved several key comparisons: (1) The proteomic profile of AH in JIA uveitis eyes was compared to controls, (2) JIA uveitis compared to other uveitis entities, (3) JIA uveitis specifically compared to silent chronic anterior uveitis, and (4) a comprehensive comparison across all four groups. They identified protein peaks in all groups, measured by mass-to-charge (m/z) ratio.

Six protein peaks at m/z 6475, 6672, 8725, 8840, 13,762, and 27,981 showed significant differences between the groups. In the JIA group, the presence and expression levels of the peak at m/z 13,762 were significantly increased compared to other uveitis entities and controls, but not compared to silent chronic anterior uveitis. Overall, JIA and silent chronic anterior uveitis samples displayed similar protein profiles

Since the peak at m/z 13,762 was most indicative for JIA and silent chronic anterior uveitis, it was purified and identified as transthyretin by LC/MS/MS. The upregulation of transthyretin was confirmed by ELISA. These findings suggested similar intraocular molecular processes in patients with chronic anterior uveitis, regardless of arthritis presence (Ayuso et al., 2013).

**References**

Ayuso, V. K., de Boer, J. H., Byers, H. L., Coulton, G. R., Dekkers, J., de Visser, L., van Loon, A. M., Schellekens, P. A. W. J. F., Rothova, A., & de Groot-Mijnes, J. D. F. (2013). Intraocular biomarker identification in uveitis associated with juvenile idiopathic arthritis. *Investigative Ophthalmology & Visual Science*, *54*(5), 3709–3720. https://doi.org/10.1167/IOVS.12-10865

Choi, J. A., Ju, H.-H. H., Lee, J., Kim, J.-E. E., Paik, S.-Y. Y., Skiba, N. P., & Rao, P. V. (2022). Increased Complement-Associated Inflammation in Cytomegalovirus-Positive Hypertensive Anterior Uveitis Patients Based on the Aqueous Humor Proteomics Analysis. *Journal of Clinical Medicine*, *11*(9), 2337. https://doi.org/https://dx.doi.org/10.3390/jcm11092337 PT  - Article

Ikeda, Y., Imai, Y., Kumagai, H., Nosaka, T., Morikawa, Y., Hisaoka, T., Manabe, I., Maemura, K., Nakaoka, T., Imamura, T., Miyazono, K., Komuro, I., Nagai, R., & Kitamura, T. (2004). Vasorin, a transforming growth factor β-binding protein expressed in vascular smooth muscle cells, modulates the arterial response to injury in vivo. *Proceedings of the National Academy of Sciences of the United States of America*, *101*(29), 10732–10737. https://doi.org/10.1073/PNAS.0404117101

Komatsu, H., Usui, Y., Tsubota, K., Fujii, R., Yamaguchi, T., Maruyama, K., Wakita, R., Asakage, M., Shimizu, H., Yamakawa, N., Nezu, N., Ueda, K., & Goto, H. (2022). Comprehensive Proteomic Profiling of Vitreous Humor in Ocular Sarcoidosis Compared with Other Vitreoretinal Diseases. *Journal of Clinical Medicine*, *11*(13), 3606. https://doi.org/10.3390/jcm11133606

Malapeira, J., Esselens, C., Bech-Serra, J. J., Canals, F., & Arribas, J. (2011). ADAM17 (TACE) regulates TGFΒ signaling through the cleavage of vasorin. *Oncogene*, *30*(16), 1912–1922. https://doi.org/10.1038/ONC.2010.565

Sepah, Y. J., Velez, G., Tang, P. H., Yang, J., Chemudupati, T., Li, A. S., Nguyen, Q. D., Bassuk, A. G., & Mahajan, V. B. (2020). Proteomic analysis of intermediate uveitis suggests myeloid cell recruitment and implicates IL-23 as a therapeutic target. *American Journal of Ophthalmology Case Reports*, *18*, 100646. https://doi.org/10.1016/J.AJOC.2020.100646

Tao, Q., Wu, L., An, J., Liu, Z., Zhang, K., Zhou, L., & Zhang, X. (2024). Proteomic analysis of human aqueous humor from fuchs uveitis syndrome. *Experimental Eye Research*, *239*. https://doi.org/10.1016/j.exer.2023.109752

Zhang, Y., Deng, Y., Jing, S., Su, G., Li, N., Huang, Z., Zhang, W., Chen, Z., Yang, P., Y., Z., Y., D., S., J., G., S., N., L., Z., H., W., Z., & Z., C. (2024). Proteomic profiling of aqueous humor-derived exosomes in Vogt-Koyanagi-Harada disease and Behcet’s uveitis. *Clinical Immunology (Orlando, Fla.)*, *259*, 109895. https://doi.org/https://dx.doi.org/10.1016/j.clim.2024.109895 PT  - Article
